# High Surface Area Ceria for CO Oxidation Prepared from Cerium *t*-Butoxide by Combined Sol–Gel and Solvothermal Processing

**DOI:** 10.1007/s10562-013-1162-8

**Published:** 2013-12-17

**Authors:** Jingxia Yang, Liliana Lukashuk, Hao Li, Karin Föttinger, Günther Rupprechter, Ulrich Schubert

**Affiliations:** Institute of Materials Chemistry, Vienna University of Technology, Getreidemarkt 9, 1060 Wien, Austria

**Keywords:** Ceria, Cerium butoxide, Sol–gel processing, Solvothermal treatment, CO oxidation

## Abstract

**Abstract:**

CeO_2_ was synthesized by combined sol–gel and solvothermal processing of gels obtained from acetaldoximate-modified cerium(IV) *t*-butoxide in the presence of the non-ionic surfactant Pluronic F127. The use of cerium(IV) *t*-butoxide as precursor contrasts very favorably with the often used ceric ammonium nitrate and results in more reliable and tailorable properties of the final materials. The kind of post-synthesis treatment of the gels and the composition of the precursor mixture proved to be crucial for obtaining high surface area ceria with a high Ce^3+^ proportion. Calcination in air or under nitrogen was compared with solvothermal treatment in ethanol or water and a combination of solvothermal treatment and calcination. The obtained materials are composed of 3.5–5.5 nm ceria nanoparticles. The highest specific surface area of 277 m^2^/g was obtained after solvothermal treatment, and 180 m^2^/g when solvothermal treatment was followed by calcination in air to remove residual organic groups. The highest Ce^3+^ proportion was 18 % after solvothermal treatment in ethanol and additional calcination in air. CO oxidation on selected samples indicated that activity scaled with surface area and thus was largest for samples solvothermally treated in ethanol. The reaction rate of the best sample was about 75-times larger than that of commercial ceria.

**Graphical Abstract:**

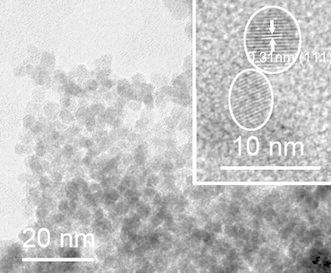
.

## Introduction

Ceria (CeO_2_) has received attention in environmental catalysis because of its high oxygen storage-release capacity associated with the Ce^4+^/Ce^3+^ redox cycle [1]. It was suggested that nano-scaled ceria with abundant oxygen vacancies would further enhance the catalytic activity [2, 3].

Various methods have been employed to synthesize CeO_2_, including the sol–gel method [4]. The latter has many advantages because the materials composition and texture can be influenced by the proper choice of precursors and processing conditions. Inorganic cerium salts, such as Ce(NO_3_)_3_·6H_2_O [5–9] CeCl_3_·7H_2_O [10] or ceric ammonium nitrate (NH_4_)_2_[Ce(NO_3_)_6_] (CAN) [11], were mainly used as precursors for sol–gel processing of ceria (including the citrate-gel route) because they are commercially available. Qi et al. [12] found that the choice of the precursor [Ce(NO_3_)_3_ and (NH_4_)_2_Ce(NO_3_)_6_] influenced the structure, surface state, reducibility and CO oxidation activity of Cu doped CeO_2_ materials.

Cerium alkoxides, Ce(OR)_3_ or Ce(OR)_4_, are less often employed as precursors because they are not easily available commercially and more reactive to humidity. Nevertheless, metal alkoxides have often advantages because the materials textures and properties can be easily modified by varying the reaction parameters. Cerium alkoxides were used, for example, to prepare ceria thin films [13, 14] or bimetallic oxides [15–17].

Apart from the precursor, the post-synthesis treatment of the gels is also an important parameter influencing the materials properties. Gels obtained by sol–gel processing are often heat-treated in air, sometimes also in N_2_ or a combination of both, to remove residual organic groups. Recently, a combined sol–gel and solvothermal process was applied for the preparation of titania which possessed a special microstructure with high surface area [18, 19].

In the work reported here, the influence of different precursors (CAN and cerium(IV) *t*-butoxide (Ce(O*t*Bu)_4_), CeB) and of different post-synthesis treatment of the gels on the morphology and microstructure of ceria was investigated. Furthermore, the catalytic activity of various CeO_2_ samples for CO oxidation was tested, with the specific surface area (S_BET_) and the Ce^3+^ (surface) concentration being very important parameters.

In previous work on high surface area titania, some of us had shown that modification of the metal alkoxide with acetaldoxime (AO) resulted in materials with superior properties, especially when combined with the non-ionic surfactant Pluronic F127 as a pore-forming agent [20]. The impact of both precursor modification and surfactant-assisted sol–gel processing on the materials properties, especially porosity and surface area, was discussed there in detail. We have therefore used AO and F127 also in this work. Substitution of metal alkoxides by bidentate ligands (such as AO) slows down the reaction rates and results in additional porosity/surface area upon calcination of the gels. Although the oximate-modified cerium alkoxide precursor was prepared in situ according to Eq. 1, it is reasonable to assume that the composition and structure of the precursors is the same as that of oximate-substituted zirconium alkoxides, Zr(OR)_4−x_(O–N=CRR′)_x_ [21], given the general similarity of Zr and Ce alkoxides.

1 $$ {\text{Ce(OR)}}_{4} + \text{x}\;{\text{RR}}'{\text{C}}\! \!=\!\!{\text{N}}\!\!-\!\!{\text{OH}}\; \to \;{\text{Ce(OR)}}_{{4 - {\text{x}}}} ({\text{O}}\!\! -\!\! {\text{N}}\!\! = \!\!{\text{CRR}}')_{\text{x}} \; + \;{\text{x}}\;{\text{ROH}} $$

## Experimental

All solvents were dried by standard methods, and all experiments involving metal alkoxides were carried out under moisture- and oxygen-free argon using standard Schlenk or glove box techniques. (NH_4_)_2_[Ce(NO_3_)_6_] was obtained from Alfa-Aesar and used as received. Commercial CeO_2_ powder from Aldrich was used for comparison.

### Synthesis of Cerium *t*-Butoxide

Cerium(IV) *t*-butoxide (Ce(O*t*Bu)_4_, CeB) was synthesized according to the literature from (NH_4_)_2_[Ce(NO_3_)_6_] (CAN) and sodium *t*-butoxide in 1,2-dimethoxyethane [22, 23]. The oily residue obtained after removal of the solvent was used directly for sol–gel processing without further purification. X-ray photoelectron spectra (XPS) proved that the sample contained no residual sodium from the preparation.

### CeO_2_ Synthesis

**Scheme 1:**
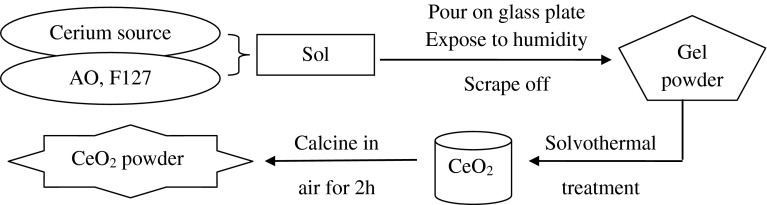
Schematic synthesis protocol for CeO_2_ by combination of sol–gel and solvothermal processing

Sols were prepared from CeB, AO, F127 and 1,2-dimethoxyethane (DME) in a 1:2:0.005:40 ratio. In some reactions no F127 was used. In a typical experiment, 5 mmol of CeB was dissolved in 10 mL of DME, then 10 mmol of AO was added and the mixture was stirred for 30 min. After addition of 0.025 mmol of F127 the mixture was stirred for another hour. No water was added at this stage. The sols were then deposited onto glass sheets (20 × 30 cm^2^), which had been cleaned with 10 % NaOH, *i*PrOH and acetone and dried at 100 °C. The as-deposited films were exposed to ambient humidity at room temperature for 24 h. The obtained solids were then scraped off with a razor blade to get a gel powder and directly treated by one of the following methods:

- Holding at 500 °C in air for 2 h in an Al_2_O_3_ crucible; heating rate 2°/min (AC).
- Solvothermal treatment (ST) at 200 °C for 6 h (ST) in a 60 mL autoclave with 30 mL solvent (gel prepared from 5 mmol of CeB). Two different solvents were used, viz. EtOH (STE) and H_2_O (STH).
- ST, followed by calcination as described for AC (ST-AC) (Scheme 1).

The labeling of the samples is as follows: (i) precursors (CeB, AO and/or F127), (ii) post synthesis treatment (AC, STE, STH). For example, CeB/AO/F127-STE-AC is a sample prepared from a mixture of CeB, AO and F127, followed by ST in ethanol (STE) and calcination in air (AC).

### Characterization

Thermogravimetric analysis (TGA) was performed on a Netzsch Iris TG 209 C in a platinum crucible in synthetic air with a heating rate of 10 °C/min. Infrared spectra (IR) were recorded on a Bruker Tensor 27 working in ATR Micro Focusing MVP-QL with a ZnSe crystal, using OPUS software version 4.0 for analysis.

X-Ray powder diffraction (XRD) measurements were performed on a Philips X’Pert diffractometer using Cu-K_α_ radiation (λ = 1.5406 Å). Scanning electron micrographs (SEM) were obtained on a Quanta 200 (FEI) equipped with a Genesis (EDAX) energy dispersive spectrometer, with a voltage of 7.5 kV. XPS were measured on a Specs XPS system (XR 50 Mg/Al-Dual Anode, PHOIBOS 150 hemispherical analyzer).

High resolution transmission electron micrographs (HRTEM) were recorded on a TECNAI F20 operated at 200 kV. Before the measurements, the samples were ultrasonically dispersed in EtOH for 30 min, and then deposited on copper grids covered with carbon films.

Nitrogen sorption measurements were performed on an ASAP 2020 (Micromeritics). The samples were degassed in vacuum at room temperature for at least 5 h prior to measurement. The total surface area was calculated according to Brunauer, Emmett and Teller (BET), and the pore size distribution (from the desorption branch) according to Barrett, Joyner and Halenda (BJH).

### Catalytic Activity

The CO oxidation reaction was performed in a continuous-flow fixed-bed quartz reactor under atmospheric pressure. An amount of 20 mg of each sample was loaded into the reactor and pretreated with synthetic air (30 mL/min) at 200 °C for 40 min (heating rate 10 °C/min). Then the sample was cooled to 30 °C in flowing synthetic air, and a mixture of 5 vol% CO, 10 vol% O_2_ and 85 vol% He (total flow 50 mL/min) was introduced. The system was then heated to 650 °C with a ramping rate of 5 °C/min. The concentrations of CO and CO_2_ in the outlet streams were monitored by gas chromatography with a HP-PLOT Q column and a flame ionization detector.

CO temperature-programmed reaction (CO-TPR) over ca. 20 mg samples were also performed in a continuous-flow fixed-bed quartz reactor under atmospheric pressure. The catalyst samples were pre-treated with synthetic air at 200 °C for 40 min (heating step 10 °C/min) at a flow rate of 30 mL/min. After cooling to room temperature the samples were exposed to a mixture of 5 vol% CO and 95 vol% He (total flow 50 mL/min) at room temperature. Then the system was ramped up to 900 °C at a heating rate of 10 °C/min. The gas stream was analysed by an online quadrupole mass spectrometer (QMS) (Prisma Plus QMG 220, Pfeiffer Vacuum) equipped with a Faraday detector.

## Results and Discussion

### Gel Preparation

Sols were prepared from CeB (or CAN), AO, F127 and 1,2-dimethoxyethane (DME) in a 1:2:0.005:40 ratio. In some reactions no F127 was added to check the influence of the surfactant. Gelation was induced by exposure to ambient humidity at room temperature. The obtained solid gels were then treated by different methods, viz. calcination in air (AC), ST in EtOH (STE) or H_2_O (STH), or ST, followed by calcination (ST-AC) (see Sect. 2.2).

When (NH_4_)_2_[Ce(NO_3_)_6_] (CAN) was used, the onset temperature was about 230 °C and the gel decomposed completely below 300 °C with a weight loss of about 72 %. This indicates that part of the material was lost during the TGA measurements due to deflagration. It should be pointed out that samples obtained from CAN sometimes exploded upon calcination. The solid obtained from CAN after sol–gel processing was water-soluble; thus no network had been formed with ambient moisture. The exothermic decomposition of CAN and the solubility of the obtained gel render CAN unappealing as precursor. For this reason CAN-derived samples will no longer be considered in the following.

In contrast, when CeB was used as a cerium precursor, the onset temperature in TGA was about 495 °C, and decomposition was complete at about 600 °C, with 22 % weight loss. The X-ray diffractogram after calcination (sample CeB/AO/F127-AC) showed that the sample was phase-pure ceria (PDF-No. 34-0394) with a particle size of 9.2 nm, as calculated by the Scherrer equation based on the strongest reflection at 28.6°.

We also tested whether thermolysis at 500 °C in N_2_ for 3 h, followed by calcination in air for 2 h would result in significantly different materials properties. The particle size was only slightly smaller (8.6 nm) and the specific surface area (see below) slightly higher. For this reason, only calcination in air was used in the following.

### Influence of Different Post-synthesis Treatments

The results of BET measurements of the gels obtained from CeB and treated differently after gelation are summarized in Table 1. The AC sample had N_2_ sorption isotherms between type IV and III according to the IUPAC definition [24], corresponding to meso-/macro-porous materials. Table 1 shows that for AC calcination, the specific surface area (S_BET_) was only 8.6 m^2^/g without F127 and 12.8 m^2^/g with F127, i.e. it was not too much changed by the presence of F127 in this system. The specific surface area is too small to be interesting for catalytic applications.

**Table 1:** BET results for CeB:AO = 1:2 samples with different post-synthesis treatment

F127	Calcination	S_BET_ (m^2^/g)^a^	D_BJH_ (nm)^b^
–	AC	8.6	7.0
+	AC	12.8	12.1
+	STE	277.0	3.4
+	STH	133.6	5.5

^a^S_BET_: BET surface area, error ±5 % (from repeated experiments)

^b^D_BJH_: BJH desorption pore diameter, ±0.5 nm (from repeated experiments)

**Fig. 1:**
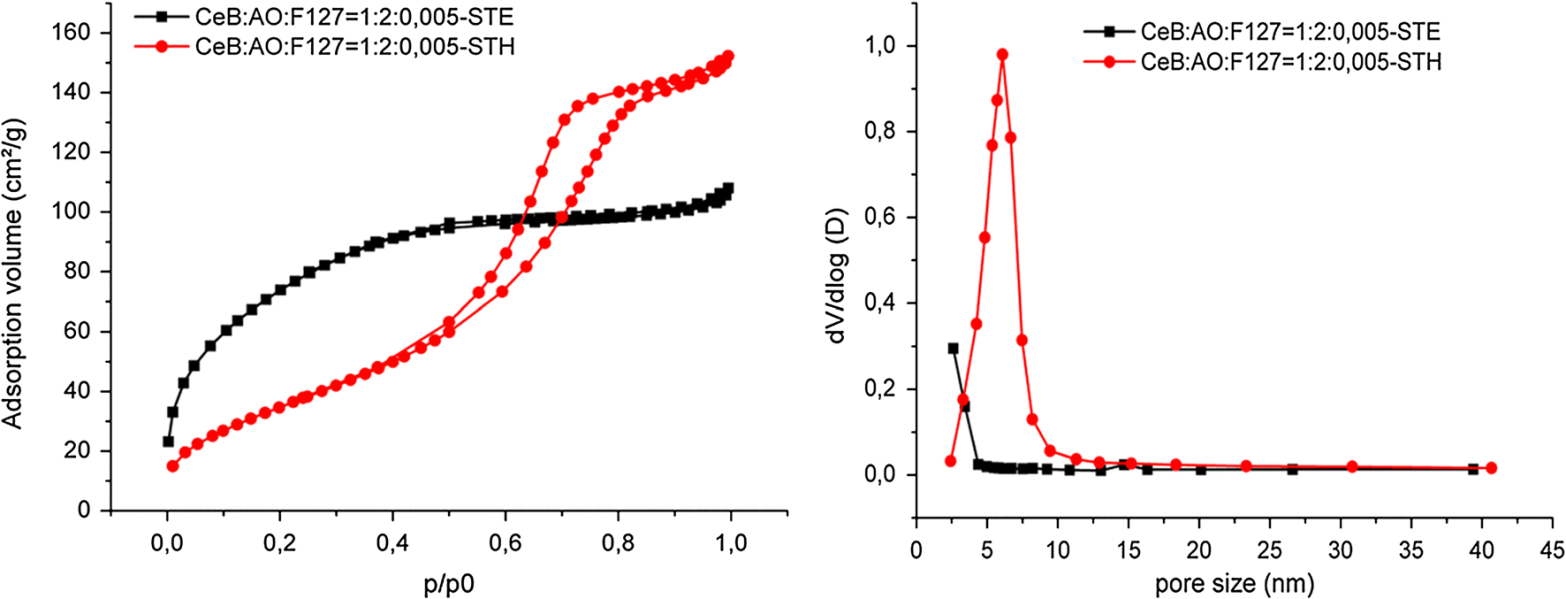
N_2_ adsorption–desorption isotherms (*left*) and pore size distribution (*right*) for different sample after ST

Because standard calcination did not provide samples with high surface areas, ST was tested, and both EtOH and H_2_O were used as ST solvent (samples labeled STE and STH, respectively). The N_2_ sorption isotherms and pore size distributions of CeB/AO/F127-ST are shown in Fig. 1, and the derived parameters are summarized in Table 1. The shape of the isotherms showed that CeB/AO/F127-STH is mesoporous with a specific surface area of 133.6 m^2^/g, while CeB/AO/F127-STE is microporous with a very high surface area of 277.0 m^2^/g. The surface area of the materials can thus be strongly improved by ST treatment, and the used solvent had a significant influence on the surface area and especially the pore size distribution (Fig. 1 right).

### Influence of the Sol Composition

After evaluation of the best post-synthesis treatment, the influence of the sol composition on the materials properties was investigated in more detail. To this end, the following experiments were performed:

- CeB: only CeB in DME in the ratio CeB:DME = 1:40.
- CeB/AO: CeB and AO in DME in the ratio of CeB:AO:DME = 1:2:40.
- CeB/F127: CeB and F127 in DME in the ratio CeB:F127:DME = 1:0.005:40.
- CeB/AO/F127: CeB, AO and F127 in DME in the ratio CeB:AO:F127:DME = 1:2:0.005:40.

The samples were solvothermally treated (STE or STH), with all other preparation parameters being the same.

**Fig. 2:**
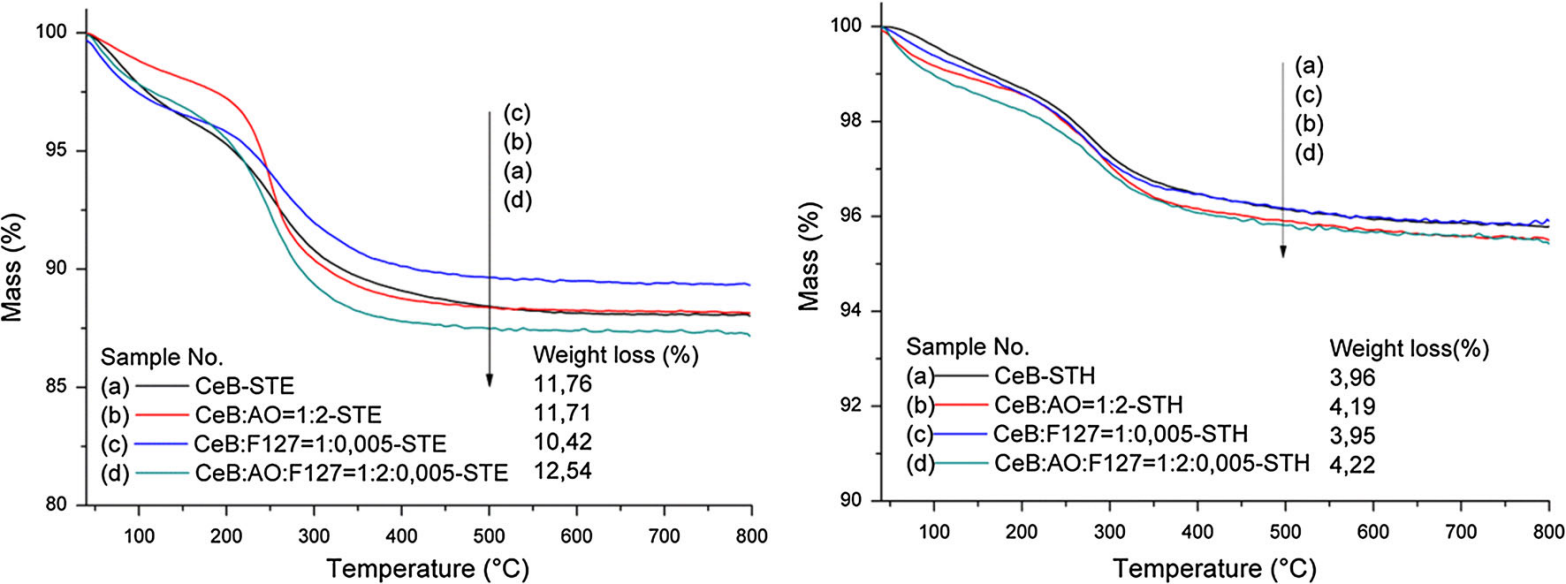
TGA curves for different CeB samples treated by STE (*left*) or STH (*right*)

TGA measurements indicated that all samples still contained organic groups after ST, as expected. The weight loss of the samples treated in EtOH was almost the same (11.5 ± 1.0 %, Fig. 2, left). When the solvent was changed to H_2_O, however, the weight loss was much lower (4.0 ± 0.2 %, Fig. 2, right). It can be concluded that during *hydro*thermal treatment, the organic constituents attached on the gel particles are already cleaved to a much larger extent than in the case of ST in ethanol.

**Fig. 3:**
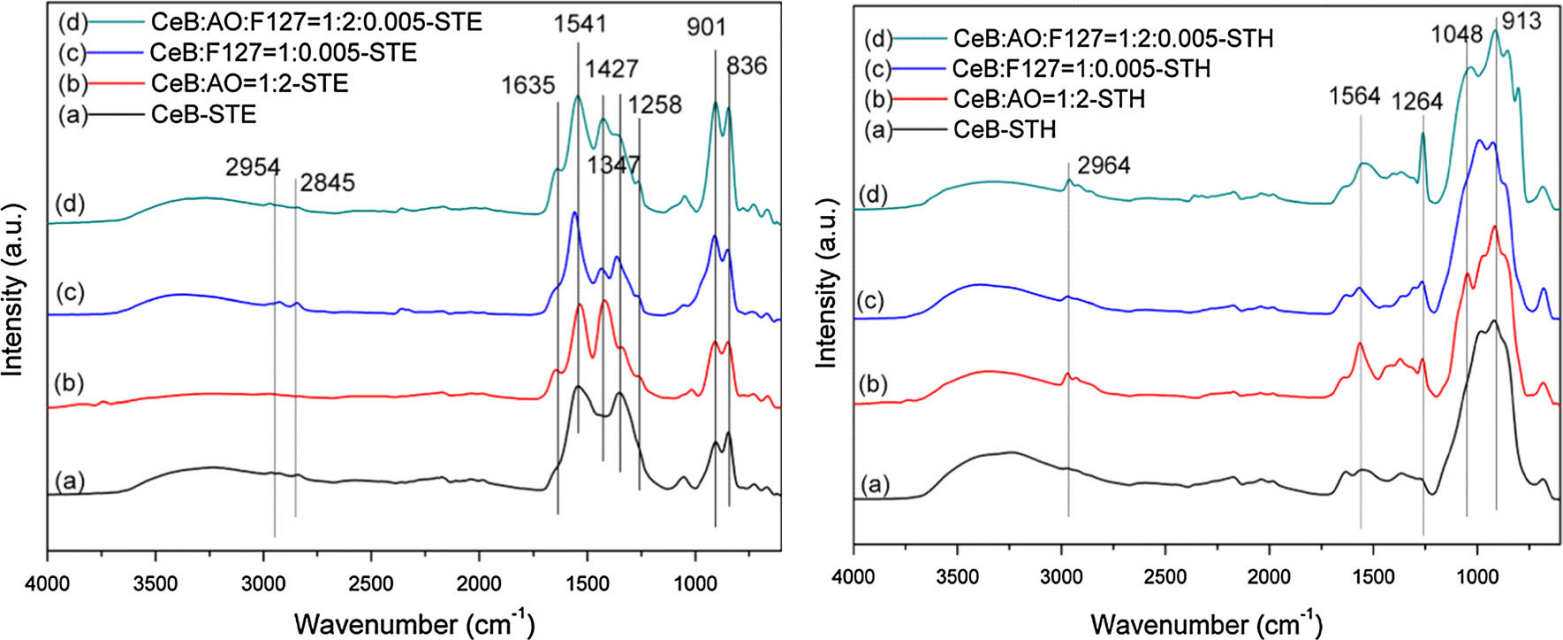
IR spectra of CeB samples treated by STE (*left*) or STH (*right*)

This conclusion was confirmed by IR measurements. When EtOH was used as a solvent (Fig. 3 left), all spectra showed strong bands at about 1300–1650 cm^−1^ (C–O, C=O, COO) and 800–1000 cm^−1^ (Ce–O–Ce), and weak bands at 2954 (CH_3_) and 2845 cm^−1^ (CH_2_). The strong bands at 1635, 1541 and 1258 cm^−1^ indicated the presence of larger proportions of carboxylate groups. The appearance of such groups on the surface of ceria nanoparticles after ST in alcohols was reported by Slostowski et al. [25]. When H_2_O was used as solvent (Fig. 3, right), only strong bands at about 1000 cm^−1^ were observed. Generally, the fundamental stretching bands (Ce–O) for CeO_2_ materials are in the range 400–500 cm^−1^ [26]. Thus, the observed bands at 800–1000 cm^−1^ can be attributed to the overtones. They may slightly shift depending on the surface groups. The weak bands at 1564 and 1264 cm^−1^ indicated that there was only a small carboxylate proportion.

The results of N_2_ sorption measurements are given in Table 2. For the STE samples the surface areas were in the following order: CeB/AO/F127-STE (277 m^2^/g) > CeB/F127-STE (247 m^2^/g) > CeB/AO-STE (210 m^2^/g) > CeB-STE (193 m^2^/g). When H_2_O was used as solvent, however, the surface area was CeB/F127-STH (181 m^2^/g) > CeB/AO-STH (167 m^2^/g) > CeB-STH (146 m^2^/g) > CeB/AO/F127-STE (133 m^2^/g). Except CeB/AO/F127-ST, the sequence was similar for both series. The surface areas for the STE samples were about 50 m^2^/g higher than that of STH samples, which may be due to the residual organic groups during solvothermal processing with EtOH, as reported earlier for ceria [25] and also indicated by the IR spectra (Fig. 3, left). The organic groups therefore appear to stabilize smaller particles and a higher porosity, and thus result in higher surface areas.

**Table 2:** BET and XRD results summary of samples prepared by ST and ST-AC treatment

ST solvent	Sol composition	ST series		ST-AC500 °C series		
		S_BET_ (m^2^/g)^a^	D_BJH_ (nm)^b^	S_BET_ (m^2^/g)^a^	D_BJH_ (nm)^b^	P_XRD_^c^ (nm)
EtOH	CeB	193.3	4.5	22.4	5.0	4.9
	CeB/AO	210.5*	3.2*	92.4*	3.1*	4.3
	CeB/F127	246.9	6.2	153.3	5.3	3.9
	CeB/AO/F127	277.0*	3.4*	88.9*	3.9*	4.0
H_2_O	CeB	146.6	3.8	143.3	3.7	3.6
	CeB/AO	167.1	5.7	165.8	5.6	4.4
	CeB/F127	181.3	4.6	178.5	4.7	4.4
	CeB/AO/F127	133.6*	5.5*	132.4*	5.4*	5.3

^a^BET surface area; error ±5 %

^b^BJH desorption pore diameter; ±0.5 nm for STE series, ±0.1 nm for STH series

^c^Crystallite particle size calculated by Scherrer equation from XRD

* Samples were synthesized at least two times, and the average values are given here

**Fig. 4:**
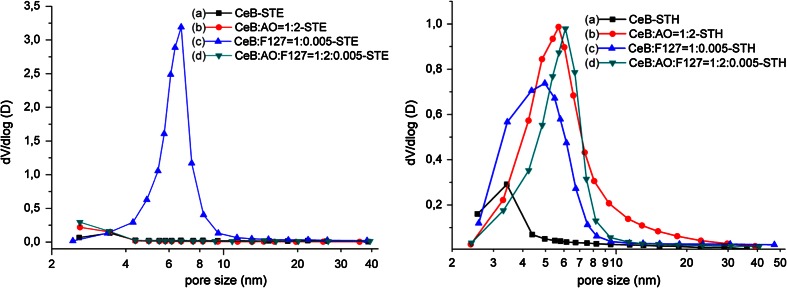
Pore size distribution for different samples after STE (*left*) and STH (*right*) treatment

When EtOH was used, only CeB/F127 had a very narrow pore size distribution in the mesoporous range (Fig. 4). When H_2_O was used, all samples except CeB-STH had almost the same pore size distribution, with the narrowest for CeB/AO/F127. The use of AO for modification of the starting cerium alkoxide had different influence on the pore size and the specific surface area when different ST solvents were used.

### Calcination After Solvothermal Treatment

**Fig. 5:**
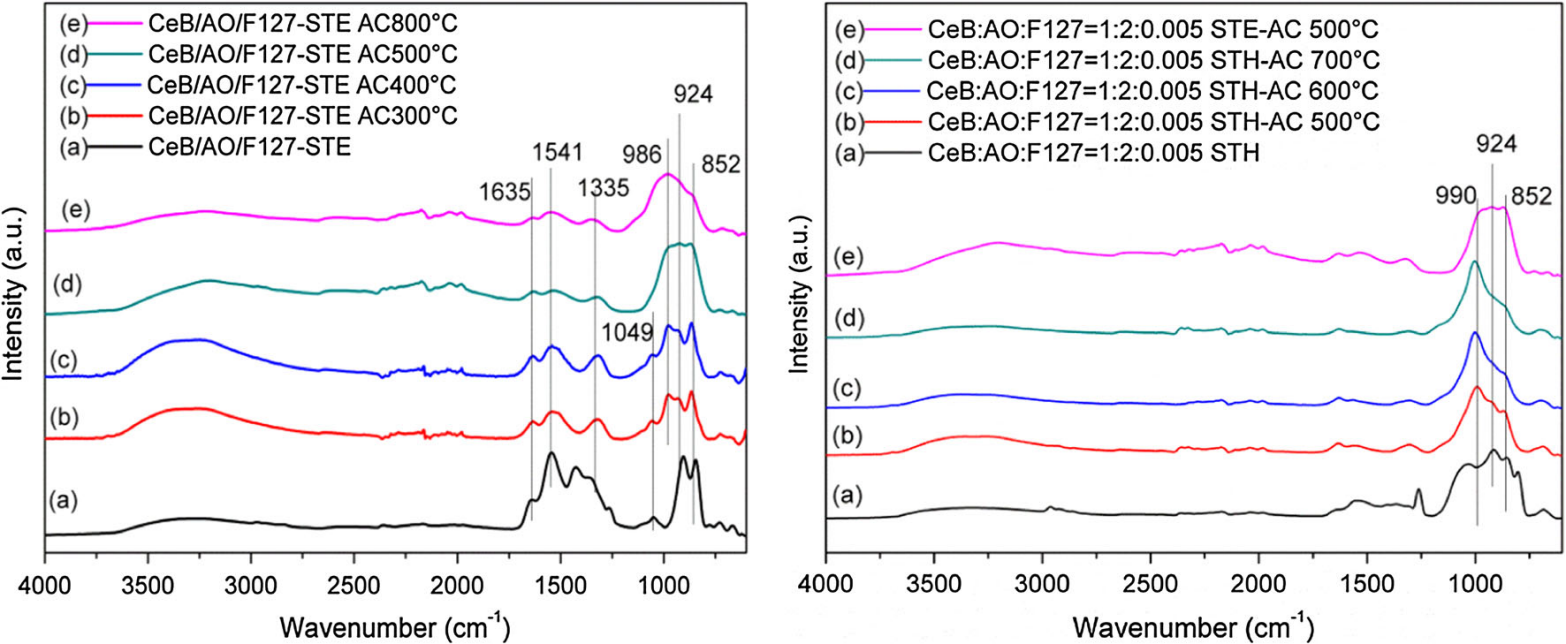
IR spectra of CeB/AO/F127-STE (*left*) and CeB/AO/F127-STH (*right*) calcined in air at different temperatures

The previous experiments had shown that residual organic groups were present after ST, especially in ethanol. To check how removal of the residual organic groups would influence the properties of the materials, selected samples were calcined in air after ST. The IR spectra of CeB/AO/F127-STE after calcination at different temperatures are shown in Fig. 5, left. The carboxylate groups were almost completely decomposed after heat treatment at 300 °C, as already indicated by the TGA measurements (Fig. 2), and only small residues remained after temperature treatment above 500 °C. The Ce–O–Ce region had a new band at 986 cm^−1^ after calcination at 300 °C. This band became stronger with increasing temperature, while the original bands at 924 and 852 cm^−1^ were only slightly reduced.

The changes of CeB/AO/F127-STH were similar to that of the STE samples (Fig. 5, right). Significant differences between the STE-AC and the STH-AC samples were, however, that the new band at 990 cm^−1^ of the STH-AC sample became much stronger during calcination than that of the STE-AC sample, while the original bands at 924 and 852 cm^−1^ and other small bands became weaker.

The experiment indicated that calcination in air at 500 °C removes most of the residual organic groups without causing excessive sintering of the materials. For the following experiments, the samples were therefore treated at 500 °C.

BET measurements after calcination (Table 2) showed that the surface area and average pore size of the STH samples were not significantly changed. In contrast, the surface area of the STE-AC samples was significantly reduced compared with the corresponding STE samples while the average pore sizes only changed slightly. For example, CeB/AO/F127-STE had a surface area of 277 m^2^/g and an average pore size of 3.4 nm, but the corresponding values of CeB/AO/F127-STE-AC changed to 88.9 m^2^/g and 3.9 nm, respectively. The change of the surface area of the STE samples can possibly be attributed to the higher proportion of organic constituents present after ST and their removal upon AC. The surface area of the STE samples after calcination is in each case significantly lower than that of the STH samples, while the average pore diameters are in the same range.

The XRD pattern after calcination (Table 2) showed that all samples were nanocrystalline CeO_2_ with similar particle sizes in the range 3.5–5.5 nm (calculated by the Scherrer equation based on the strongest peak at 28.6°). Interestingly, this was only half the size than that of the particles obtained by calcination only, i.e. without ST. For comparison, we also analyzed the particle sizes of two samples after ST prior to calcination. The average particle size of CeB/AO/F127-STE was <3 nm (4.0 nm after calcination) and that of CeB/AO/F127-STH 5.1 nm (5.3 nm after calcination). The crystallite size of the final material is thus determined by the ST and is not significantly changed when the residual surface groups are removed upon subsequent calcination.

A possible explanation for the different particle sizes (and surface areas) between the AC and STE-AC or STH-AC series is that dissolution-deposition equilibria under solvothermal conditions determine the particle size. Similar results were found for ceria solvothermally treated in different alcohols [25]. The obtained particles are capped by residual (organic) groups which are then removed upon calcination. Only crystallite sizes can be determined by XRD, but no information is obtained about agglomeration/aggregation of the crystallites. The lower BET surface area in the STE series (see Table 2) may thus be due to a more pronounced agglomeration/aggregation of the nanocrystals. In contrast, when the initially obtained gels were calcined without prior ST, removal of the organic groups and mass transport coincide and the formation of larger particles is thus favored.

**Fig. 6:**
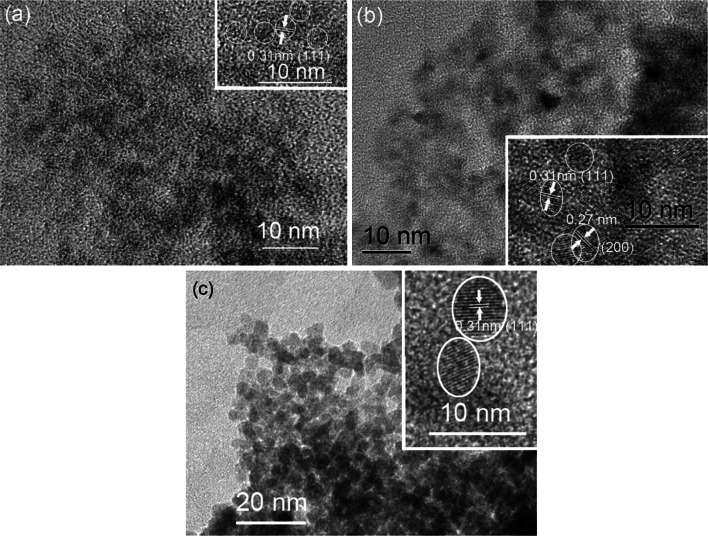
HRTEM images of sample **a** CeB/AO/F127-STE, **b** CeB/AO/F127-STE-AC and **c** CeB/AO/F127-STH-AC. The *insets* are higher magnifications of the same image

In order to get more information on the influence of calcination, CeB/AO/F127-STE, CeB/AO/F127-STE-AC and CeB/AO/F127-STH-AC were characterized by HRTEM (Fig. 6). Only (111) lattice fringes with a distance of 0.31 nm were observed for the STE and STH-AC samples, while the (200) lattice fringes, with 0.27 nm distance, were also observed for STE-AC.

### Catalytic Properties

**Fig. 7:**
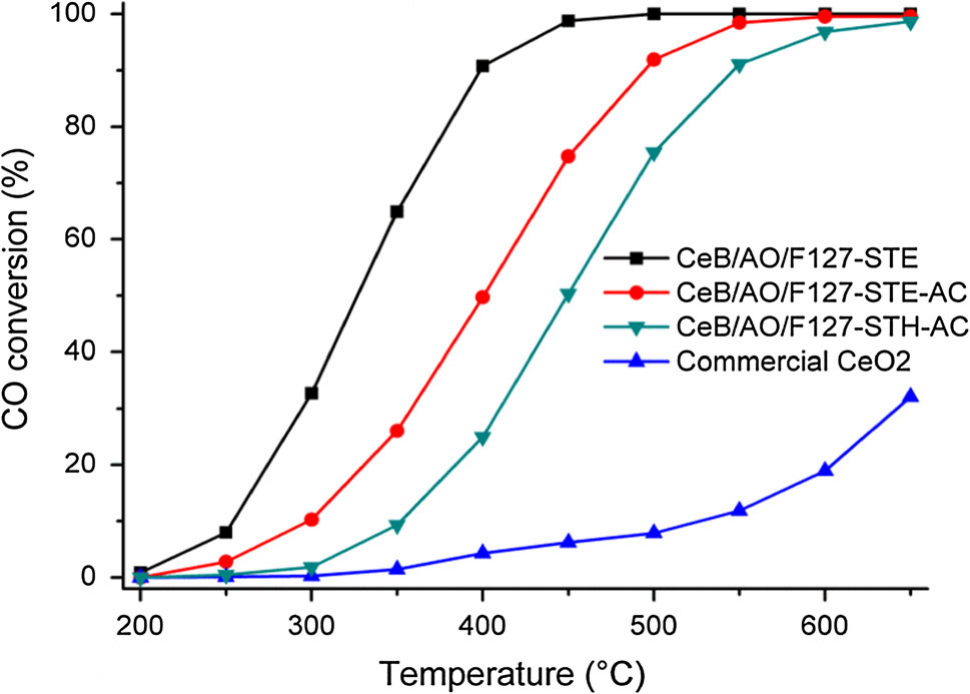
CO oxidation at different temperatures for CeO_2_ samples (20 mg), compared with commercial CeO_2_

The catalytic activity for CO oxidation was tested for three samples, i.e. CeB/AO/F127-STE, CeB/AO/F127-STE-AC and CeB/AO/F127-STH-AC (Fig. 7), with commercial CeO_2_ serving for comparison. The ignition temperature (T_10 %_, the reaction temperature required for 10 % CO conversion) of commercial CeO_2_ is 528 °C and the light-off temperature T_90 %_ (the temperature at which 90 % conversion of CO is achieved) is ≫650 °C at the conditions specified in the Experimental Section. Both T_10 %_ and T_90 %_ are much lower for the three samples prepared in our work (Table 3); the order is CeB/AO/F127-STE < CeB/AO/F127-STE-AC < CeB/AO/F127-STH-AC.

**Table 3:** Properties of different ceria samples (20 mg) for CO oxidation

Sample	P_XRD_ (nm)^a^	S_BET_ (m^2^/g)^b^	D_BJH_ (nm)^c^	Ce^3+^ (%)^d^	T_10 %_ (°C)^e^	T_90 %_ (°C)^f^	R_250 °C_ (mol/s m^2^)^g^	r_250 °C_ (mol/s g)^h^
CeB/AO/F127-STE	<3	277.0	3.4	12	253	398	2.32 × 10^−8^	6.42 × 10^−6^
CeB/AO/F127-STE-AC	4.0	88.9	3.9	18	298	494	2.52 × 10^−8^	2.24 × 10^−6^
CeB/AO/F127-STH-AC	5.3	132.4	5.4	12	353	546	2.75 × 10^−9^	3.64 × 10^−7^
Commercial CeO_2_	57	1.5	16.8	8*	528	>>650	5.76 × 10^−8^	8.63 × 10^−8^

^a^Crystallite particle size calculated by Scherrer equation from XRD

^b^BET surface area; error ±5 %

^c^BJH desorption pore diameter; ±0.5 nm for STE series, ±0.1 nm for STH series

^d^Ce^3+^ ratio from XPS, error ±2

^e^Reaction temperature for 10 % CO conversion

^f^Reaction temperature for 90 % CO conversion

^g^Normalized specific reaction rates of CO oxidation on a unit surface area at 250 °C

^h^Reaction rate of CO oxidation at 250 °C/g

* Value taken from Ref. [1]

For a better comparison of the catalytic activity of the different samples, the normalized reaction rates at 250 °C per gram (r_250 °C_) and per unit surface area (R_250 °C_) are also shown in Table 3. The sequence of r_250 °C_ is CeB/AO/F127-STE > CeB/AO/F127-STE-AC > CeB/AO/F127-STH-AC > commercial CeO_2_. The reaction rate at 250 °C (r_250 °C_) of the best sample CeB/AO/F127-STE is about 75-times larger than that of commercial CeO_2_, that at 300 °C about 125-times larger.

The CO oxidation activity of the STE and STE-AC catalysts correlates with the specific surface area (Table 3). About three times higher surface area of STE produces about three times higher rates. This can be seen from comparing the reaction rate per surface area, i.e. the amount of CO converted on a unit surface area of the catalyst at 250 °C (R_250 °C_), which are basically identical. Accordingly, when the specific activities of the two catalysts CeB/AO/F127-STE and CeB/AO/F127-STE-AC are compared based on weight, the rates r_250 °C_ (reaction rate of CO oxidation at 250 °C/g) show an about threefold difference. Commercial ceria roughly fits into this sequence; the specific surface area is about 180-times smaller than that of CeB/AO/F127-STE, which reduces r_250 °C_ by a factor of 75.

The third sample CeB/AO/F127-STH-AC exhibits a specific surface area somewhat larger than CeB/AO/F127-STE-AC but shows lower rates (both R_250 °C_ and r_250 °C_) by a factor of ten. This must be due to other parameters besides the surface area. In the literature different effects have been made responsible for activity differences of ceria. For example, the crystallite size of CeO_2_ is known to be a crucial factor for CO oxidation because size may affect the abundance of oxygen vacancies and thus of associated Ce^3+^ ions. Smaller crystallite sizes induce a higher fraction of oxygen vacancies due to lower barrier of vacancy formation [27, 28]. One of the proposed mechanisms for CO oxidation on CeO_2_ is oxidation by lattice oxygen of CeO_2_ with subsequent creation of oxygen vacancies (and Ce^3+^) which in turn induces formation of active oxygen species [29, 30].

**Fig. 8:**
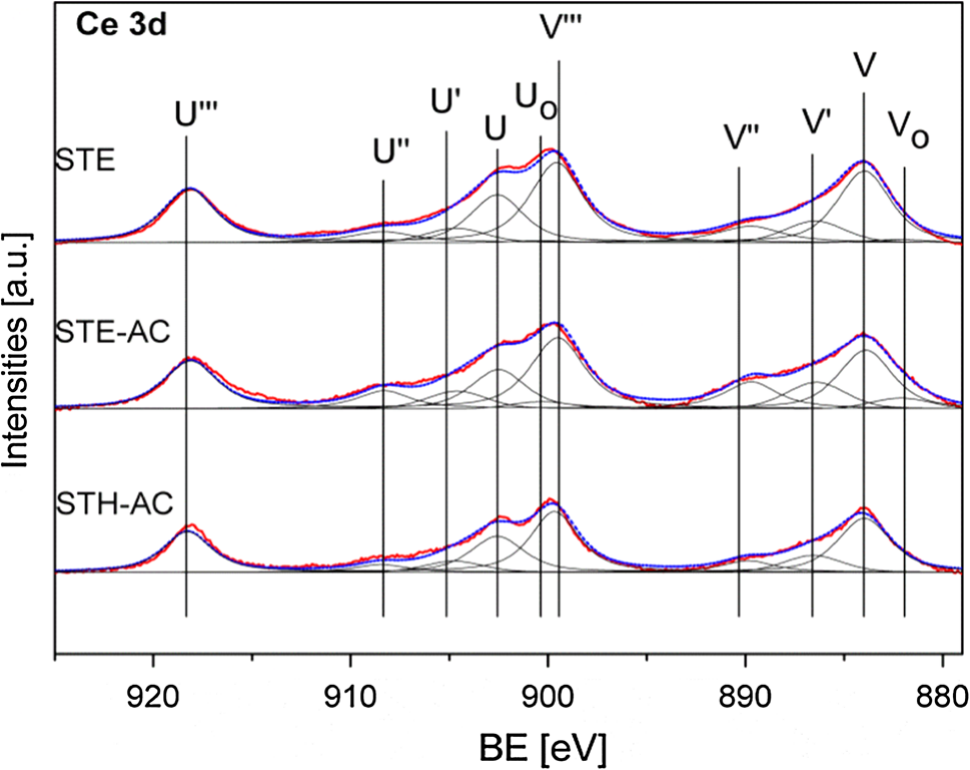
XP spectra of the Ce 3d region for CeB/AO/F127-STE, CeB/AO/F127-STE-AC and CeB/AO/F127-STH-AC (*red* raw spectrum, *blue* peak sum after fitting, *black* fitted peaks)

In order to evaluate the Ce^3+^ proportion, XP spectra were recorded to characterize the three samples (see Fig. 8; Table 3). The Ce 3d region is composed of several doublets (v denotes Ce 3d5/2, u denotes Ce 3d3/2). Specifically, v_0_, v′, u_0_, u′ belong to Ce^3+^ species, whereas v, v′′, v′′′, u, u′′ and u′′′ belong to Ce^4+^ species [31]. In order to estimate the Ce^3+^/(Ce^3+^+Ce^4+^) ratio, the relative peak area of the u_0_ (v_0_) and u′(v′) with respect to the area of the entire Ce 3d region was determined [12]. The results (Table 3) indicate that the Ce^3+^ proportion can be influenced by the synthesis parameters to some extent. When CeB/AO/F127-STE-AC and CeB/AO/F127-STH-AC are compared, the use of EtOH for solvothermal processing results in a higher Ce^3+^ proportion. This may be due to the fact that EtOH is reductive and can be oxidized to acetaldehyde or carboxylate [25]. Besides the solvent, the post-synthesis treatment can also influence the Ce^3+^ proportion. While CeB/AO/F127-STE had only 12 % Ce^3+^, the Ce^3+^ proportion of CeB/AO/F127-STE-AC increased to 18 % when calcined in air. One possible explanation is that decomposition of the carboxylate group might require some oxygen from the ceria, causing oxygen deficiency.

Nevertheless, a correlation between the Ce^3+^ proportion and the specific catalytic activity was not observed. It may explain the difference between the specific catalytic activity of the STH-AC and STE-AC samples. On the other hand, however, STE and STH-AC have a comparable Ce^3+^ proportion but STE has one magnitude higher catalytic activity.

**Fig. 9:**
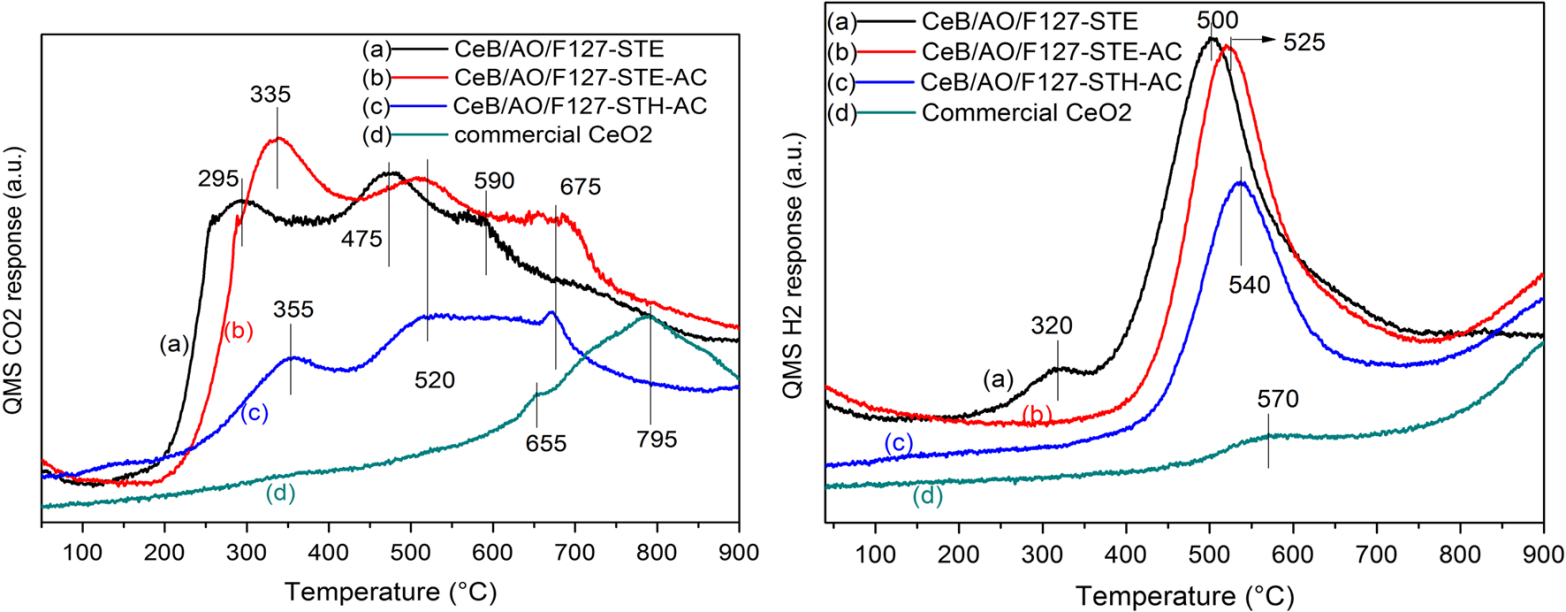
CO_2_ (*left)* and H_2_ (*right*) evolution during CO-TPR over different CeO_2_ samples (20 mg)

In CO temperature programmed reaction (CO-TPR) experiments, CO_2_ and H_2_ evolution were observed when the samples were exposed to CO and heated to 900 °C (Fig. 9). The CO_2_ evolution showed three peaks for all samples, i.e. <400, 450–600, and >650 °C. In the literature, these features were attributed to the removal of surface lattice oxygen, water gas shift between CO and surface OH groups (CO + OH → 1/2 H_2_ + CO_2_), and extraction of bulk oxygen, respectively [32]. Interestingly, the low temperature CO_2_ peak of STE is shifted by around 50 °C to low temperature relative to the STE-AC and STH-AC samples. In addition, STE also exhibits, besides the main H_2_ peak at 500–570 °C (which is due to the water gas shift reaction), an additional feature at around 320 °C, which was not observed for the other samples. The H_2_ evolution at low temperature for STE could be attributed to reactions involving the residual organic group after the synthesis. This would also explain the shift of CO_2_ evolution to lower temperature for STE.

As discussed above, CO oxidation by ceria depends on the oxygen vacancies (Ce^3+^), but the surface OH can also contribute to the reaction. The same catalytic activity of STE and STE-AC may be due to the compensation of the Ce^3+^ proportion (which is lower for STE) and the influence of highly reactive OH groups resulting from the residual organic species in the STE sample. STE-AC and STH-AC do not contain such OH groups, as the organic species were largely removed during the calcination. Therefore, despite the missing OH groups, the specific activity exhibited by STE-AC is the result of high Ce^3+^ content. A possible explanation for the different specific catalytic activity may thus be the different CeO_2_ surface states formed in the different post-synthesis treatments, and the reductive ability of EtOH might be a key factor.

## Conclusions

We have shown in this work that the use of cerium alkoxides as sol–gel precursor for ceria has clear advantages compared with (NH_4_)_2_[Ce(NO_3_)_6_]. Gels were prepared from acetaldoximate-substituted cerium *t*-butoxide in the presence of the surfactant F127. The latter served to create interparticle porosity after calcination. However, the post-synthesis treatment of the obtained gels had a larger influence on the resulting specific surface area than the composition of the starting mixture. ST in either ethanol or water resulted in materials with distinctly higher specific surface areas and smaller crystallite size than by calcination alone. Specific surface areas of up to 277 m^2^/g were obtained which is unprecedented for ceria (commercial ceria ranges around 2 m^2^/g). Post-synthesis calcination of the gels allows removing of the residual organic groups. Both the solvent used for ST and the calcination process can influence the Ce^3+^/Ce^4+^ ratio. The highest Ce^3+^ proportion was 18 % for CeB/AO/F127-STE-AC.

Tests of CO oxidation on selected samples indicated that a high catalytic activity is related to a high surface area and the surface groups created by the post-synthesis treatment. With regard to the latter, ST of the samples in ethanol proved to be particularly efficient. The sample with highest surface area (277 m^2^/g) from ST with ethanol outperformed commercial ceria by a factor of 75.
